# Commentary on: Divalent metal cofactors differentially modulate RadA-mediated strand invasion and exchange in *Saccharolobus solfataricus*

**DOI:** 10.1042/BSR20230058

**Published:** 2023-06-28

**Authors:** Irene C. Mangialavori

**Affiliations:** Universidad de Buenos Aires, Consejo Nacional de Investigaciones Científicas y Técnicas (CONICET), Instituto de Química y Fisicoquímica Biológicas Dr. Alejandro Paladini (IQUIFIB), Facultad de Farmacia y Bioquímica, Junín 956, Ciudad Autónoma de Buenos Aires C1113AAD, Argentina

**Keywords:** ATPASE ACTIVITY, CALCIUM, ssoRadA, STRAND EXCHANGE

## Abstract

RecA ATPases are a family of proteins that catalyzes the exchange of complementary DNA regions via homologous recombination. They are conserved from bacteria to humans and are crucial for DNA damage repair and genetic diversity. In this work, Knadler et al. examine how ATP hydrolysis and divalent cations impact the recombinase activity of *Saccharolobus solfataricus* RadA protein (ssoRadA). They find that the ssoRadA-mediated strand exchange depends on ATPase activity. The presence of Manganese reduces ATPase activity and enhances strand exchange, while calcium inhibits ATPase activity by preventing ATP binding to the protein, yet destabilizes the nucleoprotein ssoRadA filaments, allowing strand exchange regardless of the ATPase activity. Although RecA ATPases are highly conserved, this research offers intriguing new evidence that each member of the family requires individual evaluation.

The recombinase ssoRadA belongs to the RecA ATPases, a family of proteins that play a crucial role in DNA damage repair and genetic diversity. The central reaction catalyzes by these recombinases is the exchange via homologous recombination of complementary DNA regions in an ATP-dependent manner. This family of proteins is conserved from bacteria to humans and includes the bacterial RecA, archaeal RadA, and eukaryotic Dmc1 and Rad51. The amino acid sequence of the RadA and the eukaryotic Rad51 and Dmc1 is highly conserved, while the bacterial RecA has structural and functional different characteristics [2]. Thus, archaeal RadA is considered a better model for eukaryotic recombinases.

The overall structure of the RecA monomer is formed by a small N-terminal domain, a catalytic domain, and a large C-terminal domain (Figure 1A). The catalytic domain includes the Walker A and B motifs, two sequences that are involved in nucleotide-binding and are conserved in ATPases and GTPases [3].

**Figure 1:**
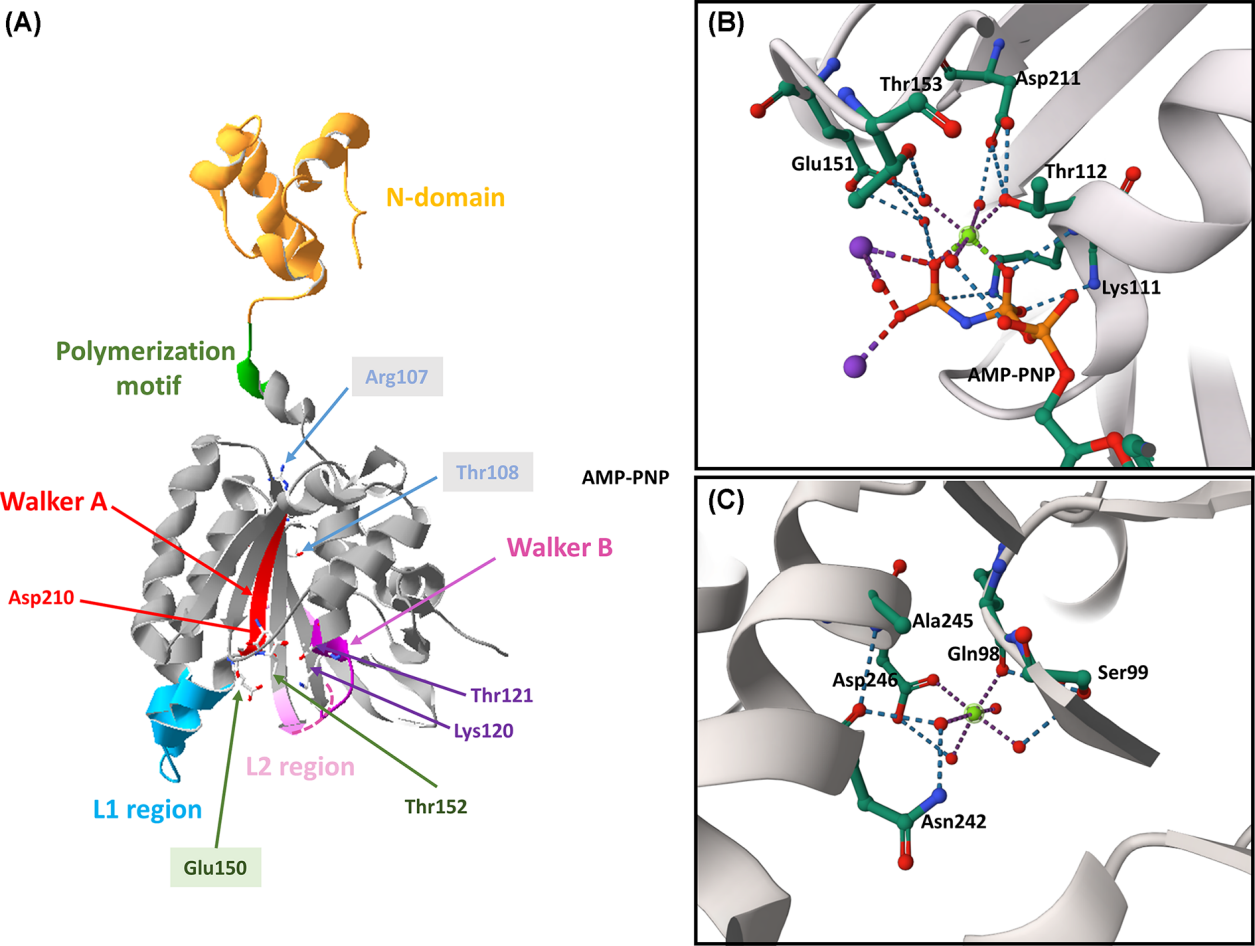
Mg binding sites in RadA ATPase (**A**) Structure of ssoRadA (PDB code: 2Z43). The polymerization motif and the L1 and L2 regions are identified in green, light blue, and pink, respectively. These regions were selected by sequences alignment between ssoRadA (Uniprot code: Q75055) with the *Methanococcus voltae* RadA (Uniprot code: Q75055) taking into account what has been reported in Wu et al. [7]. The sequences alignment (not shown) was performed with EMBOSS Needle [19]. The Met258-Val280 sequence of the L2 region was not solved (pink dashed lines).The Walker A and B motifs are identified in red and violet, respectively. In the catalytic domain, Lys120 and Thr121 (homologous to Lys 111 and Thr 112 in mvRadA); Glu 150 (homologous to Glu151), Thr152 (homologous to Thr153), and Asp 210 (homologous to Asp211) are indicated with arrows. This figure was generated using Swiss-pdbViewer [20]. (**B**) Primary Mg-binding site of mvRadA (PDB code: 2FPM). Water molecules are represented as red spheres. The Mg-ligand bonds and selected hydrogen bonds are shown with dashed violet and blue lines, respectively. The catalytic Glu151 and the main residues are shown in the figure. (**C**) Secondary Mg-binding site. The secondary Mg ion (green sphere) is coordinated by four water molecules, the main chain carbonyl of Gln98, and the side chain carboxylate of Asp246. Panels B and C were generated using Mol Viewer [21].

The functional model of these recombinases indicates that, in the presence of ATP, a single strand of DNA (ssDNA) binds to RecA, through two disordered regions (L1 and L2 motif) located in the cytoplasmic domain, forming a helical nucleoprotein filament [4]. The formation of this filament is cooperative, and nucleation requires the union of 5 or 6 protomers [5]. The RecA monomers interact through the polymerization motif, which is located between the N-terminal and catalytic domains. Magnesium-bound ATP (Mg∙ATP) is located at the interface formed by two adjacent subunits. The basic structural element of the helical filaments of yeast Rad51 and ssoRadA is a dimer, indicating that likely this is the functional unit of RecA family proteins [2]. The RecA nucleoprotein filaments move through the dsDNA looking for homology regions. Thus, the RecA filament bound to ATP and ssDNA is considered the structural intermediate responsible for homology pairing to a donor dsDNA [4]. If a homology region is found, the ssDNA invades and displaces the homologous strand in the donor dsDNA, forming a new heteroduplex or D-loop. This is known as the invasion process. This interaction between the ssDNA and the target dsDNA allows the strand exchange, and then, the ssDNA can dissociate from the nucleoprotein filament [6]. The RecA filament bound to dsDNA corresponds to the structural intermediate after the strand exchange occurred [4].

In these processes, the binding of ATP and the ATPase activity are involved to a lesser or greater extent. In the absence of ATP, the bacterial RadA proteins form compact filaments that lead to an inactive form. While in the presence of ATP, the filaments take a more extended conformation that constitutes the active form [7]. In this active form, the interaction with the dsDNA is stabilized, facilitating the invasion process and the strand exchange. Without ATP or in the presence of ADP, the dissociation of RadA after strand exchange is favored. The binding and hydrolysis of ATP require cofactors, with magnesium (Mg) being the primary ligand for ATP under normal conditions. However, other divalent [8] or trivalent [9] cations can replace Mg. The ability of an ATPase to use ATP-C^+^, where C^+^ is a cation other than Mg, as a substrate depends on subtle differences that are not always conserved within a family proteins. Knadler et al. [1] conducted a study on the archaeal ssoRadA recombinases, examining in detail the effects of Mg, manganese (Mn), and calcium (Ca) on ATPase activity, invasion process, and strand exchange.

The recombinase ssoRadA was expressed in a heterologous system as the authors described previously [10]. Here, they tested the purification process in the presence and absence of EDTA, which chelates Mg, and observed that the ATPase activity of ssoRadA from both purifications behaved differently, leading them to hypothesize that a divalent cation other than Mg could remain bound and affect protein function.

In the presence of high concentrations of ssDNA and either Mg, Ca, or Mn, the ssoRadA ATPase activity as a function of ATP increased with a saturable behavior (micaelian hyperbola). When Mg was replaced by Mn or Ca, the *K*_m_ for ATP was not significantly changed, but the maximum activity of ssoRadA was reduced by 60% for Mn and 95% for Ca. This could be due to ATP, possibly ATP-Ca or ATP-Mn under the tested conditions, does not bind to ssoRadA or, it binds but cannot be hydrolyzed. Both mechanisms have been observed in other ATPase families [9,11]. For ssoRadA, ATP was bound with a dissociation constant (*K*_D_) of approximately 1 µM in the presence of Mg and Mn. In contrast, in the presence of Ca, the *K*_D_ for ATP was higher than 800 µM. These results indicate that Ca, but not Mn, destabilizes ATP binding to ssoRadA.

RecA recombinases are proteins that promote the exchange of DNA strands through a series of steps that require ATP binding, formation of a nucleoprotein filament, homology searching, invasion, and strand exchange. Although ATPase activity is not essential for RecA, it regulates these steps [12]. To study the effect of different metal ions on the ssoRadA-mediated strand invasion, the authors used a method called the D-loop assay. This experimental strategy separates the nucleofilaments by size allowing the heteroduplex formed in the invasion process to be identified. Furthermore, experiments were carried out in the absence and presence of an ATP regeneration system to evaluate the effect of these divalent cations when the ATP/ADP ratio is high (ATPase activity is inhibited) or low. They found that replacing Mg with Ca or Mn increased strand invasion when the ATP/ADP ratio was low, but the effect was different for each divalent ion. Mn increased the formation of heteroduplexes quickly but then decreased, while Ca increased heteroduplex formation later. At each time, the amount of heteroduplex depends on a balance between its formation and disappearance rates. According to the temporal behavior in each case, replacement of Mg by Mn would increase the formation of heteroduplexes while by Ca would decrease its disappearance, i.e., Ca prevents the strand exchange. This effect is different from that observed in human Rad51 (hRad51), where Ca stimulates strand exchange [6]. In hRad51, Ca inhibits the ATPase activity leading the stabilization of the hRad51∙ATP∙ssDNA active state and the decrease of the hRad51∙ADP∙ssDNA inactive state. A similar mechanism occurs in ssoRadA in the presence of Mn. On the contrary, Ca destabilizes the interaction between ssoRadA and ATP, and between ssoRadA-ATP and ssDNA, slowing down homology checking and abolishing the requirement of ATP hydrolysis for strand exchange.

The activity of ATPase helps RecA filaments switch between active and inactive forms by binding ATP and ADP. Knadler et al. [1] used a non-hydrolyzable analog of ATP, ATPϒS, to study the effect of ATPase activity on strand exchange. The non-hydrolyzable ATP analogs have been extensively used to obtain information on the RecA family [2–4,13] and other ATPases [14–16]. In RecA, ATP interacts with the Walker A and B motifs, which are important for ATP hydrolysis. The Walker B motif has the form LhhhDE, where *h* represents a hydrophobic residue, and L, D and E correspond to leucine, aspartic and glutamic, respectively. The Asp residue coordinates the Mg ion (Figure 1B) and the Glu residue, known as the catalytic glutamic, is essential for the ATP hydrolysis. In ssoRadA (Figure 1A), the Walker B motif corresponds to the 206LIVVDS211 sequence. The Walker A (or P-loop) motif interacts with the ATP phosphate and has the form G-x(4)-GK-[TS], where *x* represents any residue, and G, K, T and S correspond to glycine, lysine, and threonine, respectively. In ssoRadA (Figure 1A), the Walker A motif corresponds to the 114GEFGSGKT121 sequence. The Lys residue, which corresponds to Lys^120^ in ssoRadA, is essential for the binding of ATP. The authors previously showed that replacing Lys^120^ with Ala (K120A) has a more dramatic effect on ATPase activity than replacing it with Arg (K120R) [17]. In this study, they found that the invasion process was differentially affected in the K120A and K120R mutants in the presence of Mg, Ca, and Mn. In the presence of Mg both mutants catalyzed the strand invasion. However, the K120R variant produced approximately 20 times more invasion products than in the presence of Ca or Mg. A high ATP:ADP ratio this variant increased the invasion process but interfered with the strand exchange. Furthermore, a high ATP:ADP ratio in the presence of Mg interfered with joint molecule formation and suppressed the conversion to the nicked circular product of the K120A mutant but had no significant effect on the K120R. Therefore, the authors suggest that K120A, but not K120R, may need to bind free ADP to successfully perform strand exchange.

Finally, Knadler et al. [1] present an interesting discussion where they compare the effect of ATPase activity and divalent cations on ssoRadA and other members of the RecA family. They suggest that there may be a second Mg-binding site in the protein based on evidence from *Methanococcus voltae* RadA (mvRadA). If Ca replaces Mg at this site, it could lead to the active nucleofilament and allow ssoRadA-mediated strand exchange even without ATPase activity.

In the structure of mvRadA with the non-hydrolyzable ATP analog AMP-PNP, two Mg ions and two potassium ions (K) were identified [7]. The Mg bound to the ATP analog is referred as ‘primary Mg’ (Figure 1B) and the other as ‘secondary Mg’ (Figure 1C). The primary Mg is located in the region that forms the interface between two subunits, while the two K ions are located near the γ-phosphate of the ATP analog. In the P-loop, the catalytic Glu151 and Asp211 (homologous to Glu150 and Asp210 in ssoRadA) participate of the Mg-binding site. The secondary Mg ion also is located in the interface between two subunits but approximately 11 Å away from the primary one. It is coordinated by four water molecules, the main chain carbonyl of Gln98, and the side chain carboxylate of Asp246 (Figure 1C). In ssoRadA, these residues are homologs to Arg107 (Figure 1A) and Glu245. The Ser99 and Asn242 residues form hydrogen bonds with the water molecules coordinating the Mg, and are homologs to Thr108 and Thr241 of ssoRadA.

In the structure of mvRadA with AMP-PNP in the presence of Ca and Mg [18], one Mg ion and two Ca ions are bound to the ATP analog (Figure 2A). The γ-phosphate of the ATP analog interacts with Lys111 and the Mg ion. One Ca ion interacts with the 2′- and 3′-hydroxyls of the bound AMP-PNP (Figure 2B). Arg296 and Gly108 (homologous to Arg295 and Gly117 in ssoRadA, respectively) form hydrogen bonds with two of the water molecules coordinating Ca. The second Ca ion replaces the two K ions (Figure 2C), and a third Ca ion replaces the secondary Mg ion in its binding site (Figure 2D). This is similar to what happens in other ATPase families where a second Mg-binding site can inhibit ATPase activity when Mg is replaced by other cations [9]. Therefore, the evidence strongly supports the hypothesis proposed by Knadler et al. [1] regarding the mechanism by which calcium destabilizes RadA filaments.

**Figure 2:**
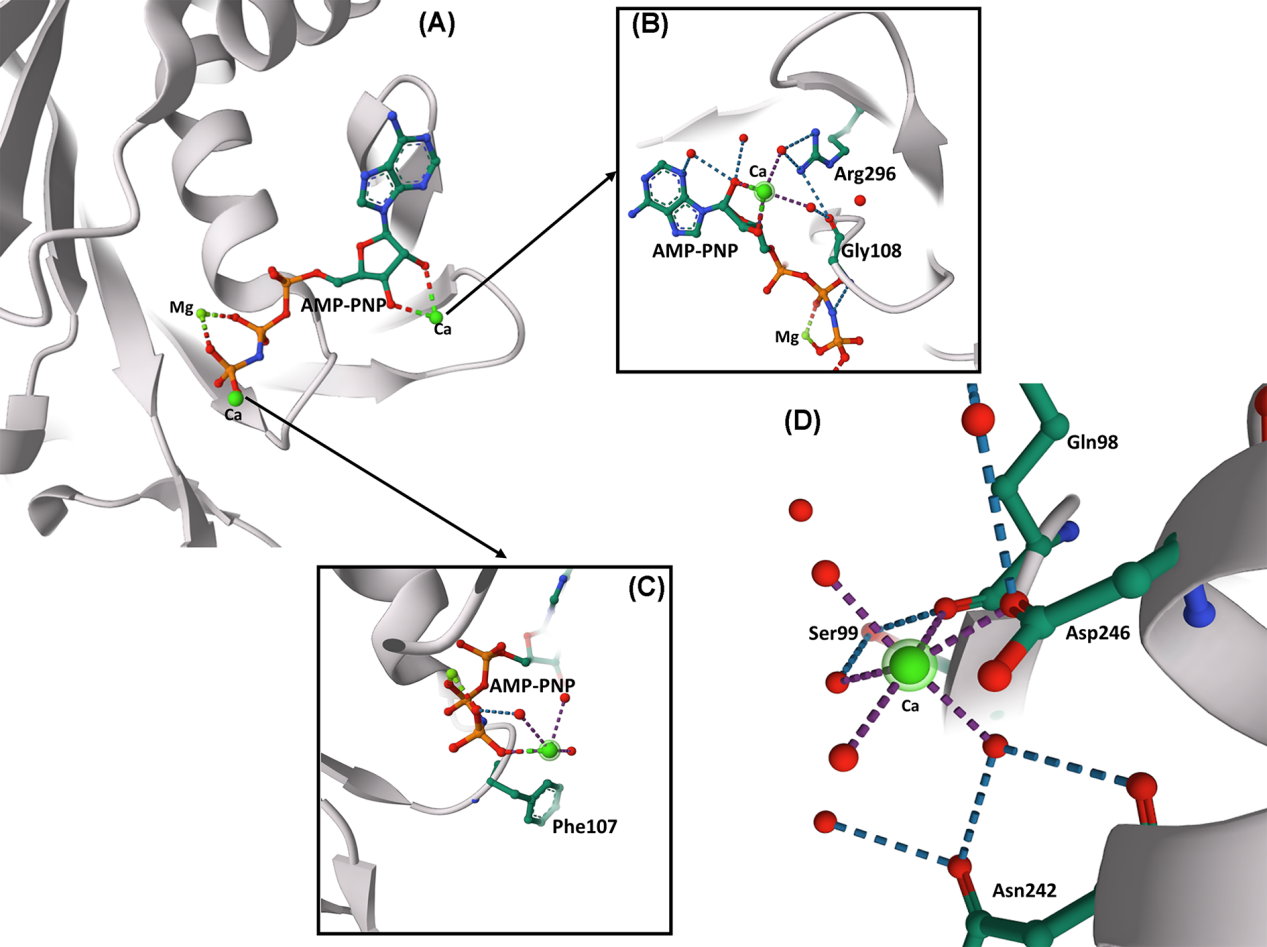
Ca-binding sites in RadA ATPase (**A**) In structure of mvRadA with AMP-PNP, Ca and Mg (PDB code: 2I1Q), one Mg and two Ca ions are bound to the ATP analog [18]. The γ-phosphate of the ATP analog interacts with Lys111 and the Mg ion. One Ca ion interacts with the 2′- and 3′-hydroxyls of the bound AMP-PNP (zoom in **B**). Arg296 and Gly108 (homologous to Arg295 and Gly117 in ssoRadA, respectively) form hydrogen bonds with two of the water molecules coordinating Ca (red spheres). The second Ca ion replaces the two K ions (zoom in **C**). (**D**) A third Ca ion replaces the secondary Mg ion in its binding site.

In summary, this study provides a detailed understanding of how divalent cations and ATPase activity affect ssoRadA function. These results are important for identifying differences between different RecA ATPases, which is essential for designing potential therapeutic strategies based on their differential inhibition.

## Abbreviations

hRad51: human Rad51
ssDNA: single strand of DNA
ssoRadA: *Saccharolobus solfataricus* RadA protein
